# Expression of Pro-Angiogenic Markers Is Enhanced by Blue Light in Human RPE Cells

**DOI:** 10.3390/antiox9111154

**Published:** 2020-11-20

**Authors:** Concetta Scimone, Simona Alibrandi, Sergio Zaccaria Scalinci, Edoardo Trovato Battagliola, Rosalia D’Angelo, Antonina Sidoti, Luigi Donato

**Affiliations:** 1 Department of Biomedical, Dental, Morphological and Functional Imaging Sciences, University of Messina, 98125 Messina, Italy; cscimone@unime.it (C.S.); salibrandi@unime.it (S.A.); asidoti@unime.it (A.S.); ldonato@unime.it (L.D.); 2 Department of Biomolecular Strategies, Genetics and Avant-Garde Therapies, I.E.ME.S.T., 90139 Palermo, Italy; 3 Department of Chemical, Biological, Pharmaceutical and Environmental Sciences, University of Messina, 98166 Messina, Italy; 4 DIMEC (Department of Medical and Surgical Sciences), University of Bologna, 40121 Bologna, Italy; sergio.scalinci@unibo.it (S.Z.S.); etbattagliola@gmail.com (E.T.B.)

**Keywords:** oxidative stress, retinitis pigmentosa, angiogenesis, choroidal neovascularization, biomarkers

## Abstract

Inherited retinal dystrophies are characterized by photoreceptor death. Oxidative stress usually occurs, increasing vision loss, and oxidative damage is often reported in retinitis pigmentosa (RP). More than 300 genes have been reported as RP causing. In contrast, choroidal neovascularization (CNV) only occasionally develops in the late stages of RP. We herein study the regulation of RP causative genes that are likely linked to CNV onset under oxidative conditions. We studied how the endogenous adduct *N*-retinylidene-*N*-retinylethanolamine (A2E) affects the expression of angiogenic markers in human retinal pigment epithelium (H-RPE) cells and a possible correlation with RP-causing genes. H-RPE cells were exposed to A2E and blue light for 3 and 6h. By transcriptome analysis, genes differentially expressed between A2E-treated cells and untreated ones were detected. The quantification of differential gene expression was performed by the Limma R package. Enrichment pathway analysis by the FunRich tool and gene prioritization by ToppGene allowed us to identify dysregulated genes involved in angiogenesis and linked to RP development. Two RP causative genes, *AHR* and *ROM1*, can be associated with an increased risk of CNV development. Genetic analysis of RP patients affected by CNV will confirm this hypothesis.

## 1. Introduction

The normal retina is organized in several tightly interconnected layers forming a unique structural and functional complex. The retinal pigment epithelium (RPE) is the basal layer and separates photoreceptors from the choriocapillaris vascular bed. RPE is a monolayer of polarized neural-crista-derived pigmented epithelial cells. On the apical side, it interacts with the outer segments of the photoreceptors, while on the basolateral surface it makes contact with Bruch’s membrane and choriocapillaris [1]. Although RPE provides nutrients for photoreceptors, it also takes part in the phototransduction process, being the site of visual pigment turn-over [2]. Moreover, RPE mediates metabolite transport between choriocapillaris and photoreceptors. Together with endothelial cells, it forms the blood–retinal barrier (BRB), a highly selective structure that regulates molecule passage from blood to retina [3]. By pumping fluid out from the subretinal space, RPE contributes to maintaining the negative hydrostatic pressure required for adhesion between RPE and photoreceptors. Cl^−^ ions enter RPE cells at the apical membrane by Na^+^/K^+^ ATPase and exit the basolateral one by Cl^−^ channels. Water transport from subretinal space to choriocapillaris is associated to this ion flow [4,5,6]. RPE also secretes several growth factors involved in the regulation of vascular endothelium homeostasis. Among these, the pigment epithelium-derived factor (PEDF, encoded by the *SERPINF1* gene) acts as an antiangiogenic molecule on choriocapillaris and exerts a neuroprotective function against hypoxia-induced apoptosis [7]. Surprisingly, somatostatin, erythropoietin (EPO) and ApoA1 are also secreted by RPE cells. In the retina, somatostatin regulates Ca^2+^ signaling, glutamate release, ions and water transport, and nitric oxide function, and acts as an antiangiogenic factor on endothelial cells [8]. EPO protects retinal cells from light-induced damage and oxidative stress by inhibiting caspase and preventing apoptosis, and up-regulates the expression of pro-angiogenic factors, such as vascular endothelial growth factor (VEGF) [9]. Other vasoactive molecules produced by RPE include thrombospondin 1, which is highly represented in Bruch’s membrane [10]. Retinal angiogenesis dysfunction can arise following oxidative stress stimuli and inflammatory cascade activation often resulting in photoreceptor degeneration [11,12]. Visual cycle reactions endogenously generate reactive oxygen species (ROS) together with lipofuscin that accumulates within RPE cells. The bis-retinoid *N*-retinylidene-*N*-retinylethanolamine (A2E) is one of the major components of lipofuscin and is synthetized from all-*trans*-retinal and phosphatidylethanolamine. Following light exposure, A2E contributes to increase ROS levels, becoming toxic to RPE. Light exposure also induces retinal neovascularization mediated by the upregulation of *VEGF*. Neo-vessels probably create anastomosis with the choroid leading to retinal atrophy due to photoreceptor degeneration [13]. Although vessel formation is enhanced in certain eye diseases, such as age-related macular degeneration (AMD) or diabetic retinopathy [14], loss of choriocapillaris is observed in the late stages of retinitis pigmentosa (RP) [15]. RP comprises a wide spectrum of retinal dystrophies characterized by progressive peripheral vision loss and night blindness due to rod degeneration and impaired RPE. Cone death and loss of central vision occur during disease progression as a consequence of both the cytotoxic activity of the degenerated rods and reduced rod-derived cone viability factors. In addition, retinal vascular attenuation and choriocapillaris atrophy occur later, following photoreceptor degeneration due to reduced metabolic demand during retinal atrophy [16]. In this context, increased levels of plasma endothelin-1 (ET-1) have been found in RP patients [17]. ET-1 is produced in RPE and its expression is stimulated by thrombin treatment [18]. Reduced oxygen demand in RP retina directly determines the increase in extracellular oxygen concentration, enhancing RPE migration [19,20]. However, RP patients occasionally develop choroidal neovascularization (CNV), an abnormal intravasation of choroidal vasculature into the RPE or subretinal tissue [21]. To date, more than 300 genes have been identified as causative of retinal dystrophies. However, molecular mechanisms leading to CNV onset have not yet been elucidated. It is well known that it may be resolved by intravitreal injection of bevacizumab, a monoclonal antibody that blocks VEGF activity [22], suggesting that CNV can arise due to an imbalance between pro-angiogenic and anti-angiogenic factors. However, there is little literature available on RP causative genes and their role in CNV development. The aim of the present study is to clarify if, following A2E and blue light exposure, CNV can be documented in specific RPE subtypes linked to specific causative gene(s). In other words, the main idea is to provide preliminary findings on the evidence that not all RP causative genes can predispose to CNV onset under stressed conditions from A2E. Therefore, we studied the expression of angiogenetic markers in RPE cells treated with A2E and prioritized dysregulated genes with those that cause RP. Although RP is an inherited disease, an increase in ROS levels due to rod death seems to play a crucial role in disease progression, promoting cone degeneration and RPE dysfunction [23].

## 2. Materials and Methods

### 2.1. Human Retinal Pigment Epithelial Cell Culture Maintenance

Human primary RPE-derived cells (H-RPE—Human Retinal Pigment Epithelial Cells, 2nd passage, Clonetics™, Lonza, Walkersville, MD, USA) were cultured in T-75 flasks with RtEGM™ Retinal Pigment Epithelial Cell Growth Medium BulletKit^®^ (Clonetics™, Lonza, Walkersville, MD, USA), with added 2% *v*/*v* fetal bovine serum (FBS), 1% penicillin/streptomycin. Maintenance conditions were set at 37 °C with 5% CO_2_. Before treatment, 4 × 10^4^ H-RPE cells/well were transferred into 96-well plates (3rd passage). Three wells were considered for each condition. A2E was added to cells, after they reached confluence. The final A2E concentration was 20 μM. H-RPE cells were then transferred to phosphate-buffered saline (PBS) supplemented with calcium, magnesium and glucose (PBS-CMG). Exposure to a tungsten-halogen source (470 ± 20 nm; 0.4 mW/mm^2^) allowed for A2E activation, mimicking cytotoxicity induced by blue light. The exposure time was 30 min. Then, cells were incubated at 37 °C up to three different time points, 3, 6 and 9 h. To compare gene expression, a negative control was obtained by irradiating H-RPE cells with blue light but not treating them with A2E (0 h_untreated). This choice was driven by the evidence that, without A2E, blue light is not sufficient to trigger oxidative damage, as is required for A2E activation.

### 2.2. Methylthiazolyldiphenyl-Tetrazolium Bromide Assay to Test Cell Viability

The methylthiazolyldiphenyl-tetrazolium bromide (MTT) assay allows one to assess cell viability by measurement of the mitochondrial-dependent reduction of MTT (Sigma-Aldrich, St. Louis, MO, USA) to formazan insoluble crystals. Following A2E treatment, 10 μL of 5 mg/mL of MTT was diluted in PBS and added to the cell medium. H-RPE cells were incubated at 37 °C for 2 h before adding 100 μL 10% SDS (sodium dodecyl sulfate) in 0.01 mol/L HCl. Cultures were incubated for 16 h and then read in a Dynatech microplate reader set to 570 nm absorbance. Untreated H-RPE cells were exposed to blue light, but A2E-untreated H-RPE cells were considered a negative control. The experiment was repeated three times.

### 2.3. RNA Extraction and Whole Transcriptome Analysis

TRIzol^TM^ reagent (Invitrogen^TM^, ThermoFisher Scientific, Waltham, MA, USA) was used to purify total RNA from H-RPE cells. RNA extraction yield was assessed at the Qubit 2.0 fluorimeter by the Qubit^®^ RNA assay kit (Invitrogen^TM^, ThermoFisher Scientific, Waltham, MA, USA). Following A2E treatment, two different time points were considered at 3 and 6 h, respectively. A2E-untreated H-RPE cells, exposed to blue light, were used as a negative control (time point: 0 h). Each time point was processed three times, obtaining nine libraries in total. A total of 1 µg of total RNA was used to generate paired-end libraries by the TruSeq Stranded Total RNA Sample Prep Kit with Ribo-Zero H/M/R (Illumina, San Diego, CA, USA). Following amplification, libraries were run on a HiSeq 2500 Sequencer (Illumina, San Diego, CA, USA), using the HiSeq SBS Kit v4 (Illumina, San Diego, CA, USA).

### 2.4. FASTQ Data Quality Control and Read Mapping 

The quality check of FASTQ data generated by sequencing run was assessed by the FastQC (v.0.11.9) (https://www.bioinformatics.babraham.ac.uk/projects/fastqc/) and the QualiMap (v.2.2.1) tools [24]. Reads having a Phred quality score < 30 were removed by the Trimmomatic (v.0.39) tool [25]. Filtered reads were mapped to the GRCh38 Human Reference Genome and the Ensembl RNA database v.99, by the Qiagen CLC Genomics Workbench v.20.0 software package (Qiagen, Hilden, Germany) (https://digitalinsights.qiagen.com/products-overview/analysis-and-visualization/qiagen-clc-genomics-workbench/).

### 2.5. Gene Expression Quantification, Differential Gene Expression (DGE) and Statistical Analysis

Aligned reads were quantified by the mapping-dependent expectation-maximization (EM) algorithm [26]. Differential gene expression (DGE) analysis was performed by the Limma R package [27]. The three different conditions were compared as described: (i) 0 h_untreated vs. 3 h_treated, (ii) 0 h_untreated vs. 6 h_treated, (iii) 3 h_treated vs. 6 h_treated. DGE was expressed as log_2_ fold change (log_2_ FC) of the gene abundance.

### 2.6. Functional Gene Annotation, Enrichment Analysis and Gene Prioritization

Differentially expressed genes (DEGs) were annotated based on the InterPro [28], Reactome [29], Human Protein Atlas [30], UniProt [31], IntAct [32], Ensembl [33] and HGNC [34] databases. Functional enrichment and gene clustering analysis were performed by the FunRich: Functional Enrichment analysis tool, according to the biological pathways [35]. A full list of dysregulated genes was inputted. Only pathways showing the Bonferroni-adjusted *p*-value < 0.05 were considered.

To identify RP causative genes that might enhance angiogenesis, prioritization analysis with the ToppGene tool [36] was performed. In detail, genes related to retinal vascular development, dysregulated in A2E-treated H-RPE cells (*VEGFA*, *PDGFB*, *CCN1*, *LRP5*, *FZD4*), were used as the “training gene set”. As the “Test gene set”, all those reported in the RetNet database (https://sph.uth.edu/retnet/home.htm) were inputted. Considered annotation terms included: “GO: Biological Process”, “Human phenotype”, “Mouse phenotype”, “Pathway”, “PubMed” and “Disease”. Only results showing the Bonferroni-adjusted *p*-value < 0.05 were considered significant.

### 2.7. Quantitative RT-PCR Data Validation

To validate observed FC values from RNA-Sequencing, quantitative real-time polymerase chain reaction (qRT-PCR) was performed. Six genes were selected and quantified at both time points (3 h_vs._0 h, 6 h_vs._0 h) (Table 1). Retrotranscription was carried out by the High-Capacity cDNA Reverse Transcription Kit with RNase Inhibitor (Applied Biosystems™, Fisher Scientific, Loughborough, Leicestershire, UK) using 1 µg total RNA for 20 µL of reaction volume. Within the reaction mix, 50 ng of cDNA, 200 nM of each specific primer and 10 µL SYBR™ Select Master Mix (Applied Biosystems™, Fisher Scientific, Loughborough, Leicestershire, UK) were added. Each reaction was repeated thrice and gene expression was measured by an Applied Biosystems^®^ 7500 Fast Real-Time PCR System (Applied Biosystems, Foster City, CA, USA) by calculating the average threshold cycle (Ct) from the values obtained for each reaction. Relative gene expression was quantified using the 2^−ΔΔCt^ method. The expression level of *β*-actin was used for data normalization.

### 2.8. Statistical Analysis

In MTT assay, the cell viability of H-RPE cells was expressed as average percentage values from three replicates for each condition, and the standard deviation was calculated.

RNA-seq runs were also replicated three times. Differential gene expression was reported as log_2_ FC, and statistical significance was assessed by the multiple t-test, considering the Bonferroni-adjusted *p*-value < 0.05 as significance threshold.

Quantitative RT-PCR reactions were repeated three times and the average threshold cycle (Ct) by the values obtained for each reaction was calculated. Expression values are reported as mean of log_2_ FC. Correlation analysis between qRT-PCR and RNA-Seq gene expression values was obtained by linear regression with IBM SPSS 26.0 software (https://www.ibm.com/analytics/us/en/technology/spss/).

## 3. Results

### 3.1. A2E Affects H-RPE Cell Viability

**Figure 1:**
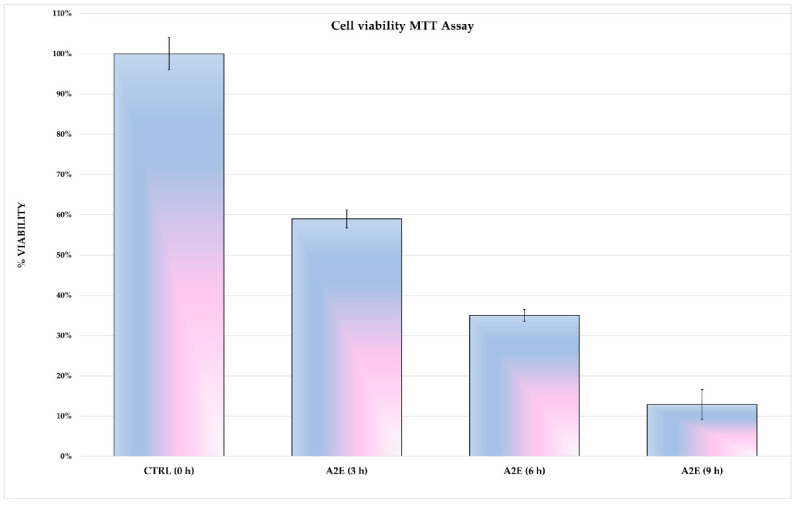
Methylthiazolyldiphenyl-tetrazolium bromide (MTT)-viability assay results. *N*-retinylidene-*N*-retinylethanolamine (A2E) had a cytotoxic effect on human retinal pigment epithelium (H-RPE) cells in a time-dependent manner. The viability of untreated (0 h) H-RPE cells and 3, 6 and 9 h A2E-exposed H-RPE cells is expressed as a percentage, as the mean ± SD (*n* = 3). Statistical significance was assessed by multiple t-tests (*p*-values < 0.05). Due to markedly decreased cell viability at 9 h with A2E, this time point was excluded in further analysis.

The cytotoxic effect of A2E and light exposure were tested in H-RPE cells. Induced cytotoxicity increased in a time-dependent manner, as shown by the cell viability trend seen in Figure 1. Cell viability was drastically decreased after 9 h of A2E and thus this time point was not considered for subsequent analysis.

### 3.2. Transcriptome Analysis and Differential Gene Expression

**Figure 2:**
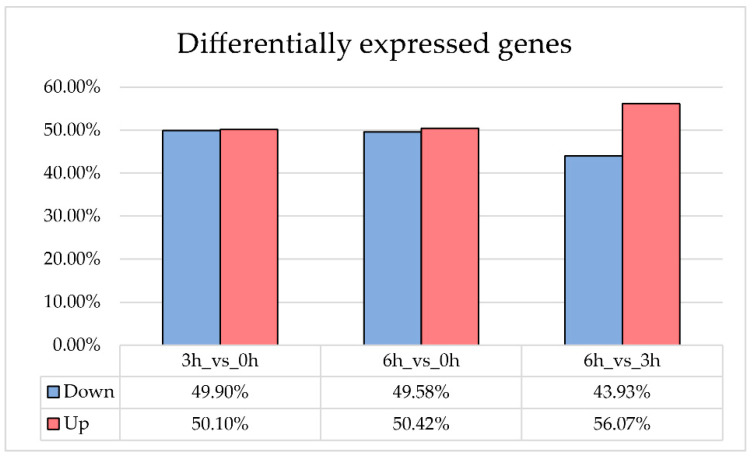
Differentially Expressed Genes (DEGs) in A2E-treated RPE cells. The histogram shows DEGs detected in RPE cells treated with A2E when compared to untreated cultures. Values are expressed in percentage. For each time point considered, blue bars indicate the down-expressed genes, while red ones refer to up-regulated genes. Both A2E-treated cells and control H-RPE cells were exposed to a blue light period.

Bulk RNA sequencing generated about 100,000,000 reads showing a mean mapping Phred quality score ≥ 30. Unique mapped reads amounted to 67.8%. DEGs were divided according to the comparison between the different time points. In detail, 3878 and 4526 genes were differentially expressed between treated and untreated H-RPE cells, respectively, 3 and 6 h after A2E and light exposure. From a comparison between the two time points after treatment (6 h vs. 3 h), 956 genes showed significative change in expression level (Figure 2) (Table S1).

### 3.3. Functional Pathway Analysis

Functional enrichment analysis clustered dysregulated genes in 1309 and 1349 pathways at 3 and 6 h after A2E treatment, respectively (Tables S2–S4). Regarding the first time point (3 h_vs._0 h), 141 pathways showed statistical significance and, of these, 19 were related to angiogenesis. Six hours after treatment (6 h_vs._0 h), the number of enriched pathways increased to 1349. However, of these, only 18 regarded the regulation of vessel morphogenesis (Tables S2–S4). Moreover, the comparison of expression values observed 6 and 3 h (6 h_vs._3 h) after A2E treatment allowed us to identify how the dysregulation of several pathways occurred in a time-dependent manner (Table 2). These include pathways related to the maintenance of endothelial cell property, such as cytoskeleton regulation and cell junction stabilization. Nevertheless, A2E treatment resulted in extra-cellular matrix (ECM) impairment by affecting β-integrin and proteoglycan signaling.

### 3.4. A2E Treatment CAUSES Loss of Retinal Blood Barrier Properties

**Figure 3:**
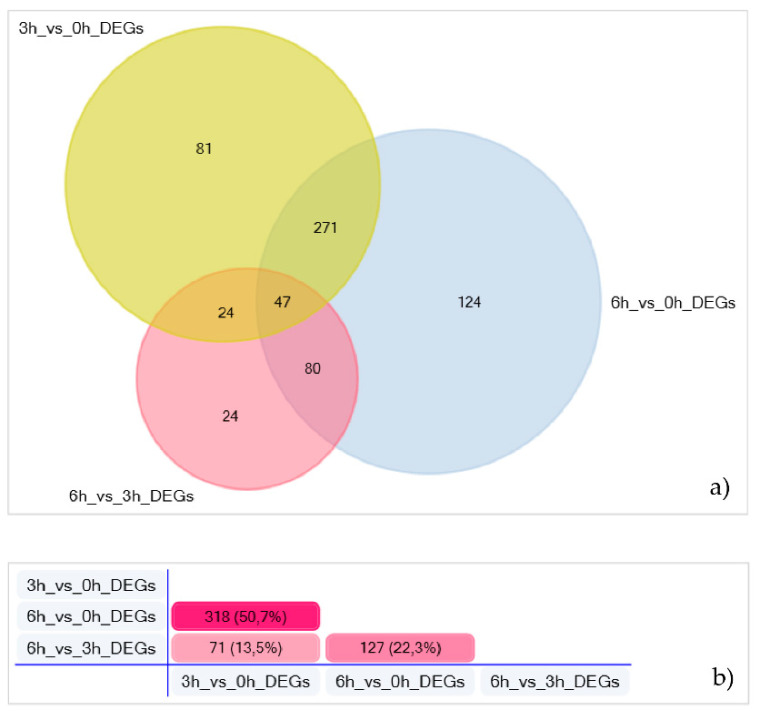
Angiogenesis-related genes dysregulated in A2E-treated H-RPE cells. (**a**) The Venn diagram reports the number of angiogenesis-related DEGs, for each time point (intersections). (**b**) Pair-comparison highlighting the percentage of DEGs between time-point pairs.

Enriched pathways related to the vessel development clustered in total 423 (3 h) and 522 (6 h) DEGs in A2E-treated H-RPE cells, when compared to untreated ones. Moreover, 175 genes were further dysregulated between 3 and 6 h, after A2E exposure. Of these, 24 were uniquely detected in this time lapse (Figure 3; Table S5).

Genes encoding for proteins involved in the maintenance of tight junction integrity (*TJP1*, *OCLN*, *ROCK1*) were slightly down-regulated at the first time point (3 h, log_2_ FC > −1), in contrast to those encoded for proteins involved in adherens zonula formation (*CAMSAP3*, *KIFC3*) that were up-regulated, suggesting that A2E can impair BRB properties, increasing its permeability. Together with the cell–cell junction, light damage also affected cytoskeleton organization due to the down-expression of genes such as *RICTOR*, which contributes to the maintenance of actin cytoskeleton polarity, *JMY*, which acts as a de novo actin filament nucleation, and *RHOA*, which regulates endothelial tube lumen extension. Moreover, *ARAP1* was up-regulated promoting epithelial–mesenchymal transition.

The expression of *TJP1* and *OCLN* further decreased after 6h of A2E treatment (log_2_ FC < −1) as well as there being a higher abundance of *CAMSAP3* and *KIFC3*, if compared to the previous time point. In addition, adherens junction formation should be enhanced by down-expression of *BMP6* and *RDX*, clustered in the “negative regulation of adherens junction organization” annotation term of the Gene Ontology database. Moreover, angiogenic markers such as *CCN1* and *VEGFA* were up-regulated in H-RPE cells 6 h after treatment, when compared to untreated ones, suggesting that continuous exposure to the blue light can promote neo-vessel formation. This finding is further supported by the increased expression of *FZD4* and *LRP5*, involved in the norrin signal pathway that contributes to retinal vasculature development.

### 3.5. A2E Treatment Impairs Expression of AHR and ROM1

Among 363 genes provided to the ToppGene tool as “Test gene set”, 57 were prioritized according to functional annotations and protein interaction networks related to angiogenesis (Table S6). Eight of these genes are known to cause different RP phenotypes (Table 3). Surprisingly, only two of these, *AHR* and *ROM1*, showed different expression values in A2E- treated H-RPE cells, when compared to untreated ones. In detail, under stress condition, *AHR* was down-expressed while *ROM1* up-regulated.

### 3.6. Quantitative RT-PCR Data Validation

**Figure 4:**
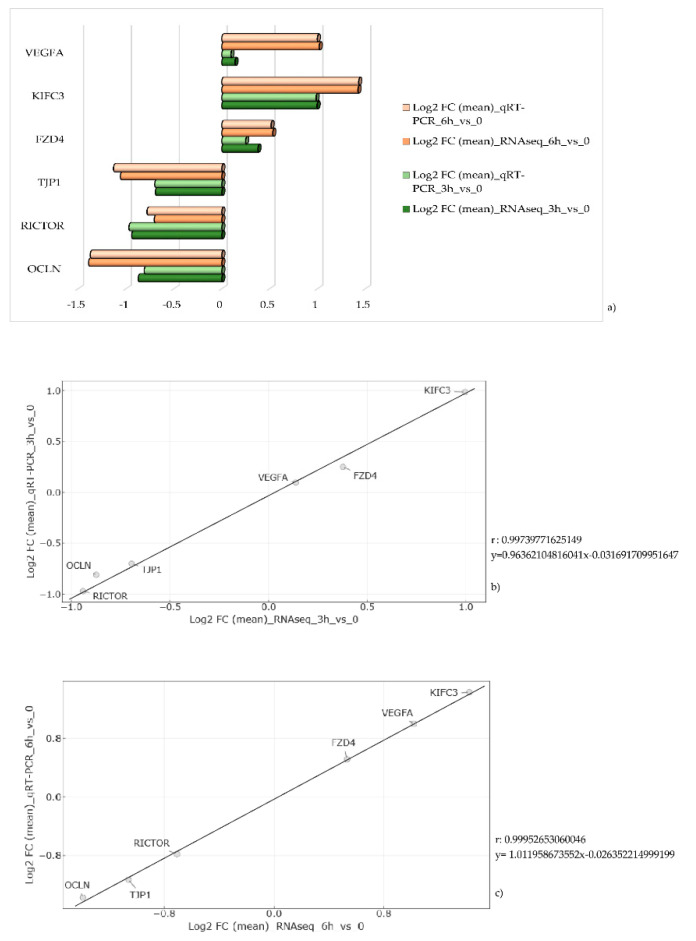
Real-time PCR results and correlation analysis. (**a**) The bar charts show a comparison between expression values (as log_2_ fold change (FC)) observed by qRT-PCR and RNA sequencing. Represented values are the average of the three replicates. (**b**,**c**) Correlation analysis between log_2_ FC observed values by RNAseq (*x*-axis) and qRT-PCR (*y*-axis) at the first (**b**) and at the second (**c**) time point.

Quantitative RT-PCR confirmed the expression values observed by RNA sequencing. The same genes were considered for both time points and data are shown in Figure 4a as the average of the three replicates. Positive correlation indexes (3 h_vs._0 h, r: 0.99739771625149; 6 h_vs._0 h, r: 0.99952653060046) were obtained by the ratio between log_2_ FC observed by RNAseq and qRT-PCR values (Figure 4b,c).

## 4. Discussion

**Figure 5:**
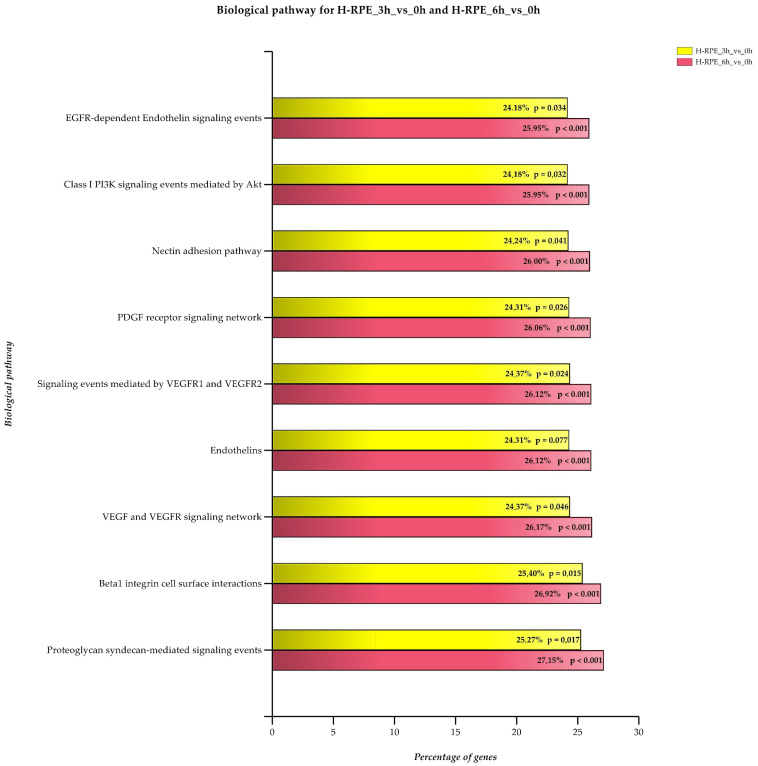
Enriched genes in biological pathways related to angiogenesis. Each biological pathway is considered both 3 and 6 h after A2E treatment. Enrichment percentage and the Bonferroni-adjusted *p*-value are reported.

The eye is continuously exposed to both natural and artificial light and this was shown to increase ROS levels within the retina. In particular, RPE and photoreceptors are extremely sensitive to oxidative damage as visual pigments, proteins and lipids are targets of peroxidation reactions. Moreover, lasting ROS exposure can trigger an inflammation cascade that results in apoptotic photoreceptor death and vision loss [45]. For this reason, oxidative stress is considered one of the main causes of AMD that usually develops in adulthood. Oxidative damage was shown to be further induced by A2E treatment and light exposure [46]. However, impairment of ROS homeostasis due to genetic mutations can lead to the onset of hereditary retinal dystrophies and, among these, RP [47]. Occasionally, RP is accompanied by a vascular event known as choroidal neovascularization (CNV). It usually onsets late, probably due to an imbalance between pro-angiogenetic and anti-angiogenetic factors. However, the reason why CNV occurs is not yet clear. With expression analysis, we investigated the role of the endogenous adduct A2E in stimulating the expression of genes involved in angiogenesis regulation. Our results showed that blue light damage occurring following A2E exposure can enhance expression of positive regulators of angiogenesis in RPE cells. RPE takes part in the formation of Bruch’s membrane that separates photoreceptors from the choriocapillaris. As previously reported, A2E treatment and light exposure increased RPE early response against oxidative damage [48]. In contrast, expression increase of genes involved in angiogenesis is not an early event and occurs very slowly, in a time-dependent manner. This supports the evidence that neovascularization happens as a complication of RP. In detail, the first events of BRB barrier damage are indicated by the dysregulation of genes involved in cell junction maintenance and in ECM remodeling (Figure 5). (The percentages and *p* values in Figure 5 must be converted into the UK/US system, i.e., 24,18 to 24.18 and *p* = 0,034 to *p* = 0.034).

A2E-treated cells showed dysregulated expression of genes clustered within nectin, focal adhesion kinase, E-cadherin and N-cadherin adhesion pathways. Our results deal with those recently reported by Ibrahim et al. [49]. The authors observed the disruption of the RPE barrier due to increased MMP2 metalloproteinase activity, following high BMP4 concentration. *BMP4* and *MMP2* were upregulated also in our samples. The high expression level of *BMP4* has been shown to stimulate *VEGFA* production [50]. Moreover, A2E induced the disruption of tight junctions, as demonstrated by the down-expression of *TJP1*, *OCLN* and *ROCK1* in treated RPE cells, when compared to untreated ones. Together with cell junctions, ECM also suffered blue light-induced stress, as shown by enrichment fold of the pathways related to β-integrin and proteoglycan signaling. Extracellular matrix remodeling in RP was linked to the increased MMP9 activity [51], while a protective role was established for its inhibitor TIMP1 [52]. *TIMP1* expression increased in H-RPE cells after A2E supplementation, and this can be considered an attempt of protection against the oxidative substance. Although ECM remodeling signaling was observed early, expression of angiogenic markers significantly changed 6h after the treatment, when endothelin, VEGF and PDGFB pathways were highly enriched. A2E treatment was shown to induce oxidative damage [46]. Under oxidative conditions, angiogenesis is enhanced due to a hypoxic condition by the activation of the PI3K/Akt cascade [53]. Despite the PI3K/Akt pathway perturbation not being described in RP, our hypothesis is that it can contribute to CNV development in patients with impaired oxidative stress defense mechanisms [54]. Moreover, the non-sudden overexpression of pro-angiogenic markers following A2E treatment can explain the reason why CNV emerges late in patients. Our results are consistent with a previous report by Vila et al., suggesting that blue light can induce vasculature remodeling in RPE cells [55,56].

Finally, we focused on RP causative genes that were dysregulated in H-RPE cells treated with A2E and that play a role in angiogenesis regulation. In this context, prioritization analysis highlighted *AHR* and *ROM1*. *AHR* encodes for the aryl hydrocarbon receptor, a transcription factor that inhibits transcription of pro-angiogenic genes. Oxidative stress reduced *AHR* expression leading to endothelial cell proliferation [57]. Moreover, in endothelial barriers, *AHR* was shown to contribute to the maintenance of barrier properties by mediating cytochrome CYP1 expression, in response to xenobiotics [58]. Taken together, these data suggest that RP patients carrying *AHR* loss of function mutations might have an augmented risk of developing CNV, as they are pro-angiogenetic genes which are no longer inhibited. Furthermore, *AHR* loss of function cannot preserve barrier integrity from continuous exposure to the cytotoxic effect of endogenous A2E.

In contrast, oxidative stress increased *ROM1* expression. It encodes for a photoreceptor that is still poorly characterized. However, heterozygous *ROM1* mutant mouse models showed narrowed retinal vasculature [59]. According to prioritization results, among all RP causative genes, here we propose that *AHR* and *ROM1* might preferably promote CNV, since they are involved in the regulation of the angiogenic process. Knowledge of the mechanisms by which these genes can impair normal vasculature remodeling needs to be elucidated. Therefore, these are preliminary results that require further investigation to establish if *AHR* and *ROM1* mutations in RP patients can act as CNV-promoting factors.

## 5. Conclusions

Retinitis pigmentosa is a large heterogenous group of inherited retinal dystrophies. To date, more than 300 genes have been linked to RP development. Although oxidative stress is commonly observed in RP patients, not all patients have a better prognosis if treated with antioxidants. Likewise, only a few RP patients develop vascular phenotypes, such as CNV. Causes that lead to CNV have not yet been elucidated. Therefore, here we propose a model of oxidative stress induced by A2E treatment and blue light exposure, on human RPE cells. Our results demonstrate that the expression of pro-angiogenic genes increases in a time-dependent manner, after treatment. ECM remodeling and cell junction impairment are key events that can contribute to increasing permeability and loss of properties of the blood–retinal barrier. Moreover, two RP causative genes, *AHR* and *ROM1*, can potentially promote CNV onset in RP patients.

## Figures and Tables

**Table 1:** Transcripts selected for RNA-seq data validation by quantitative real-time polymerase chain reaction (qRT-PCR). Regarding the data discussed in the text, 3 down-expressed and 3 up-regulated coding genes were chosen for data validation. For each gene, the HUGO (Human Genome Organization) Gene Nomenclature Committee (HGNC) name, the Ensembl-specific transcript ID, the log_2_ fold change detected by RNAseq at both 3 and 6 h after *N*-retinylidene-*N*-retinylethanolamine (A2E) treatment, the primer pair and the specific length of the amplicon are reported. FC: fold change; bp: base-pairs.

HGNC Gene Name (ID)	Ensembl Transcript ID	3 h_vs._0 h RNAseq log2 FC	6 h_vs._0 h RNAseq log2 FC	Primer Pair	Fragment Length (bp)
OCLN (8104)	ENST00000355237.2	−0.873423348	−1.391662424	ACTTCGCCTGTGGATGACTT GACCTTCCTGCTCTTCCCTT	101
RICTOR (28611)	ENST00000357387.8	−0.940830609	−0.705087984	GCTCTCTGAAGAACCTCCGA CCTGCAATCTGGCCACATTT	126
TJP1 (11827)	ENST00000356107.10	−0.694212076	−1.059176648	TCTTCGCAGCTCCAAGAGAA AGGCCTCAGAAATCCAGCTT	121
FZD4 (4042)	ENST00000531380.2	0.37699785	0.532274651	GGTTTGGTGGCCTTGTTCAA ATCACACACGTTGCAGGAAC	131
KIFC3 (6326)	ENST00000445690.7	0.995878391	1.422298598	GACCCTCACCAACGACTACA TTGCTGTTGACCTCCTCGAT	123
VEGFA (12628)	ENST00000372055.8	0.137384927	1.016674127	CTGTCTTGGGTGCATTGGAG TGATGATTCTGCCCTCCTCC	101

**Table 2:** **Enrichment pathway analysis results.** The table shows results obtained by the FunRich tool. Only pathways related to angiogenesis are reported. For each time point, the percentage of enriched genes, the fold enrichment and the Bonferroni-adjusted *p*-value are reported. Not significant: fold enrichment *p*-value ≥ 0.05.

	3 h_vs._0 h			6 h_vs._0 h			6 h_vs._3 h		
Pathway	% Enriched Genes	Fold Enrichment	*p*-Value (Bonferroni-Adjusted)	% Enriched Genes	Fold Enrichment	*p*-Value (Bonferroni-Adjusted)	% Enriched Genes	Fold Enrichment	*p*-Value (Bonferroni-Adjusted)
Beta1 integrin cell surface interactions	25.4	1.1854	0.0146	26.9	1.2561	1.0167 × 10^−7^	34.1	1.5901	1.441 × 10^−7^
EGFR-dependent Endothelin signalling events	24.2	1.1825	0.03440	25.9	1.2690	4.5939 × 10^−8^	33.6	1.645	1.3314 × 10^−8^
Endothelins	Not significant			26.1	1.26	1.3783 × 10^−7^	33.6	1.6222	4.1668 × 10^−8^
HIF-1-alpha transcription factor network	Not significant			Not significant			5.1	4.8831	8.0756 × 10^−8^
IGF1 pathway	24.2	1.1838	0.0297	25.9	1.2670	6.044 × 10^−8^	33.6	1.6424	1.5141 × 10^−8^
LKB1 signalling events	24.8	1.1964	0.0070	26.5	1.2809	4.0980 × 10^−9^	33.6	1.6210	4.4347 × 10^−8^
N-cadherin signalling events	Not significant			Not significant			8.2	2.0655	0.0252
Nectin adhesion pathway	24.2	1.1801	0.0409	26.0	1.2659	6.6551 × 10^−8^	34.1	1.6590	4.2976 × 10^−9^
PDGF receptor signalling network	24.3	1.1851	0.0257	26.06	1.2707	3.2165 × 10^−8^	33.8	1.6507	8.0949 × 10^−9^
Posttranslational regulation of adherens junction stability and disassembly	Not significant			Not significant			7.8	2.1230	0.0235
Proteoglycan syndecan-mediated signalling events	25.3	1.1846	0.0167	27.1	1.2724	7.3776 × 10^−9^	35.0	1.6389	5.2725 × 10^−9^
RAC1 signalling pathway	Not significant			Not significant			7.3	2.2556	0.0113
Regulation of CDC42 activity	Not significant			15.2	1.2478	0.0083	22.7	1.8607	1.1663 × 10^−7^
RhoA signalling pathway	Not significant			Not significant			7.3	2.2556	0.0113
Signalling events mediated by focal adhesion kinase	24.2	1.1834	0.0317	25.9	1.27	4.0022 × 10^−8^	33.6	1.6462	1.2483 × 10^−8^
Signalling events mediated by Hepatocyte Growth Factor Receptor (c-Met)	24.3	1.1851	0.0257	26.0	1.2679	5.0634 × 10^−8^	33.6	1.6398	1.7211 × 10^−8^
Signalling events mediated by VEGFR1 and VEGFR2	24.7	1.1855	0.0240	26.1	1.2705	3.0898 × 10^−8^	33.8	1.6469	9.8334 × 10^−9^
Stabilization and expansion of the E-cadherin adherens junction	Not significant			Not significant			9.3	2.1399	0.0025
Syndecan-1-mediated signalling events	24.5	1.1881	0.0178	26.2	1.2721	2.1586 × 10^−8^	33.6	1.6310	2.6854 × 10^−8^
VEGF and VEGFR signalling network	24.4	1.1782	0.0455	26.2	1.2655	5.8984 × 10^−8^	33.8	1.6368	1.6437 × 10^−8^

**Table 3:** **RP causative genes differentially expressed in A2E-treated H-RPE cells.** For each gene, the HUGO Gene Nomenclature Committee ID is indicated. The table also reports the RP phenotype linked to each locus, the role of the encoded protein in angiogenesis, and the expression values (as log_2_ FC) observed for each gene 3 and 6 h after A2E treatment. Not significant: gene expression change is not statistically significant. Not determined: no expression value detected by the analysis.

Locus (HGCN ID)	RP Phenotype	Angiogenesis	Log_2_ FC 3 h_vs._0 h	Log_2_ FC 6 h_vs._0 h
*AHR* (348)	recessive	If depleted, enhances retinal angiogenesis [37]	−0.78173956	−1.52949254
*PROM1* (9454)	recessive with macular degeneration	Expressed on EPCS; promotes neovascularization [38]	not significant	
*MERTK* (7027)	recessive	Promotes EC survival by inhibiting apoptosis [39]	not significant	
*RPE65* (10294)	recessive	Overexpressed in RPE cells surrounding CNV [40]	not determined	
*RHO* (10012)	- dominant; - recessive	If mutated, leads to retinal vessels atrophy following light exposure [41]	not significant	
*NR2E3* (7974)	- recessive in Portuguese Crypto Jews; - dominant	Regulates retinal neovascularization by targeting FLT1 [42]	not significant	
*SEMA4A* (10729)	dominant	In ECs, suppresses VEGF-mediated EC migration and proliferation by binding Plexin-D1 [43]	not significant	
*ROM1* (10254)	dominant	GO Biological process: retina vasculature development in camera-type eye (GO:0061298)	1.256611685	1.123121917
*PANK2* (15894)	recessive HARP (hypoprebetalipoproteinemia, acanthocytosis, retinitis pigmentosa, and palladial degeneration)	Required for normal development of angiogenetic properties in ECs [44]	not significant	

## Supplementary Materials

The following are available online at https://www.mdpi.com/2076-3921/9/11/1154/s1. Table S1: Differentially expressed genes; Table S2: FunRich_Report_H-RPE_3 h_vs._0 h; Table S3: FunRich_Report_H-RPE_6 h_vs._0 h; Table S4: FunRich_Report_H-RPE_6 h_vs._3 h; Table S5: FunRich_Report_DEGs_Angiogenesis; Table S6: ToppGene_Results.

## Data Deposition

According to the journal guidelines, RNA-Seq raw data were deposited in NCBI’s Sequence Read Archive (SRA) and assigned to the project identifier PRJNA622997.
